# Estimation of maximum lower limb muscle strength from vertical jumps

**DOI:** 10.1371/journal.pone.0316636

**Published:** 2025-02-27

**Authors:** Chuan-Fang Hou, Chin-Wei Hsu, Philip X. Fuchs, Tzyy-Yuang Shiang

**Affiliations:** 1 Department of Physical Education and Sport Sciences, National Taiwan Normal University, Taipei, Taiwan; 2 Department of Sport and Kinesiology, National Taiwan Normal University, Taipei, Taiwan; Instituto Politecnico de Setubal, PORTUGAL

## Abstract

Determining the one-repetition maximum (1RM) is crucial for organizing training loads, but it also is time-consuming, physically demanding, and poses a risk of injury. Vertical jumps are a less demanding and well-established method to test the ability of the lower limbs to generate great forces over a short time, which may allow for the estimation of 1RM in squatting. The purpose of this study was to develop a model for estimating 1RM back squat from ground reaction forces during vertical jumps. Thirteen healthy participants completed a 1RM back squat test, countermovement jumps, and squat jumps. Five kinematic and kinetic variables (e.g., peak and mean power, relative net impulse, jump height, and peak kinetic energy during various phases) were derived from ground reaction forces collected via a Kistler force plate (1000 Hz). Five out of 5 variables correlated with 1RM in countermovement jump and squat jump (ICC = .96–.98, *r* = .88–.95, *p* < .001 and ICC = .97–.99, *r* = .76–.90, *p* < .05, respectively). The most accurate stepwise regression model (adjusted *R*^*2*^ = .90, *SEE* =  13.24 kg, mean error =  7.4% of mean 1RM_m_, *p* < .001) estimated 1RM back squat based on peak kinetic energy during countermovement jumps. Estimation errors ranged from 7.4% to 10.7% of mean measured 1RM, with no differences between estimated and measured values (*d* <  0.01, *p* = .96–1.00). Estimating 1RM via jump tests may offer a practical alternative to traditional methods, reducing injury risks, testing intervals, and effort. Our study proposes a new possible approach for estimating 1RM back squat from jump forces, providing coaches and sports professionals with a more efficient tool to monitor and adjust training loads.

## Introduction

Muscle strength is crucial for both general physical fitness and athletic performance. Maintaining muscle strength is essential for functional health throughout various life stages, including childhood, youth, adolescence, and old age [1–3]. In children, youth, and adolescents, lower extremity muscle strength is key to motor skill development and physical fitness [4]. For the elderly, a decline in lower extremity muscle strength increases the risk of falls, frailty, and functional impairments [5,6]. In athletes, the scientific analysis and monitoring of lower extremity muscle strength are integral to their training regimes. High-quality muscle strength contributes to muscle power, rate of force development, jump height, dynamic changes of direction, and overall sports performance economy [7–10]. Additionally, functional muscle strength plays a critical role in absorbing impacts and distributing forces, which helps in preventing sports-related overuse injuries [11].

Resistance training encompassing free weights, weight machines, medicine balls, elastic tubing devices, and one’s own body weight is widely regarded as the optimal approach for enhancing muscle strength [12]. Assessing muscle strength is crucial for evaluating muscular capabilities and the designing effective strength training programs. The one-repetition maximum (1RM) back squat test serves as the traditional gold standard for assessing lower limb maximal muscle strength performance, known for its high validity and reliability [13–15]. The 1RM is also well-established in training practices as many training protocols define training loads relative to the 1RM. However, determining the 1RM can be time-consuming, physically demanding, and carries a risk of overload injuries [5,16]. Additionally, the 1RM back squat needs to be repeated frequently for monitoring purposes, which may not only increase the time and physical demands but also require adjustments to regular strength training regimens and the associated effort. Finally, 1RM testing is also associated with injury risks that can be avoided through a single jump test. The vertical jump, involving explosive extension of the hips, knees, and ankles, closely resembles the back squat movement [17]. Consequently, the vertical jump test, including the countermovement jump (CMJ) and squat jump (SJ), has been used for decades as an alternative method to assess lower limb dynamic performance. Compared to the 1RM back squat test, jump tests are more convenient, less time-intensive, impose less physical strain, and entail a lower risk of injury.

The CMJ involves a downward movement of the center of mass (COM) followed by a maximal vertical upward movement of the COM. This process, known as the stretch-shortening cycle (SSC), enhances the storage and release of elastic energy during the transition from eccentric to concentric contractions in the lower limb muscles [18]. In contrast, the SJ consists solely of an upward concentric movement of the COM, emphasizing the capacity of the lower limb muscles to produce force during a concentric-only action [19]. The CMJ includes phases of weighting, unweighting, braking, propulsion, flight, and landing. In comparison, SJ involves only the propulsion, flight, and landing phases. Additionally, ground reaction force (GRF) variables derived from these different phases of vertical jumps provide valuable insights into the characteristics of lower extremity strength, power, and fatigue [20–24]. The main categories of variables include force, velocity, power, impulse, work, kinetic energy, jump height, and countermovement depth. Previous studies have developed prediction equations that incorporate body mass alongside vertical jump height to estimate peak power in the lower extremities across diverse populations [4,25–31,]. To date, no research has investigated the estimation of 1RM back squat based on GRF variables during vertical jumps.

Power, impulse, jump height, and kinetic energy derived from GRF variables are regarded as essential metrics representing kinetics and kinematics in vertical jumps [32,33]. These metrics reflect the combined characteristics of force production, velocity optimization, and energy utilization in the lower limb muscles, which are crucial for explosive movements [34–41]. Peak power and jump height have been found to strongly correlate with 1RM back squat performance and maximal strength improvements, underscoring their relevance for assessing lower body strength, as they represent the explosive force and displacement capacity required for heavy lifts [38,40]. Additionally, mean power has been proposed as a metric for monitoring neuromuscular performance, as it directly reflects explosive strength capabilities over a sustained period, which is necessary for consistent power output during a 1RM back squat [36,41]. Relative net impulse and peak kinetic energy play essential roles in determining vertical jump performance, capturing the force exerted over time and energetic capacity during movement, which are fundamental for effective force transfer during strength exercises like the back squat [33,39]. In summary, these variables from vertical jumps represent maximal and explosive lower extremity neuromuscular capacity and may have the potential to estimate 1RM back squat performance.

Therefore, the purpose of this study was to estimate the 1RM back squat based on GRF variables (peak and mean power, relative net impulse, jump height, and peak kinetic energy) measured during CMJ and SJ. The hypotheses were as follows: 1) There will be correlations between the GRF variables of vertical jumps and 1RM back squat; 2) A predictive formula can be developed to estimate 1RM back squat from the GRF variables measured during a maximum vertical jump test.

## Materials and methods

### Participants

An a-priori power analysis (G * Power 3.1, Düsseldorf, Germany) indicated that a sample size of 11 participants would achieve the desired power of 0.80 at alpha =  0.05 to detect F-test effect sizes *f* ^*2*^ of 1.50 and higher for the main test of this study involving two predictors. The effect size *f* ^*2*^ =  1.50 is the equivalent of *R*^*2*^ =  0.60, explaining 60% of the variance. This effect size is generally considered large, which means that smaller effects may remain undetected. However, this threshold is below the expected and acceptable results reported in previous studies that developed similar estimation equations with also two predicators for related context, namely lower body power in vertical jumping (*R*^*2*^: 0.74–0.93; 2 predictors) [28,29,42,43]. Therefore, although this sample size may miss detecting small and moderate effects, it was sufficient to reliably detect the expected and practically relevant effects. The current sample size also meets the recommended requirement of a minimum sample of n =  8 for regression estimations based on low-variance samples [44], which is the case in our study. Thirteen healthy adults, actively involved in frequent strength training (2–6 times/week), participated in this study (age: 23.4 ±  0.7 years, body height: 171.6 ±  5.7 cm, body mass: 70.8 ±  10.2 kg, 1RM back squat: 1.7 ±  0.4 BW, ten males and three females, recruitment period: 2018/08/15–2018/12/15). None of the participants had engaged in weight training within 24 hours before the experiment as per the protocol by Wang et al. [45]. All participants were provided with detailed information regarding the risks and benefits associated with their involvement. The Human Research Ethics Committee of National Taiwan Normal University provided written approval of the study (approval number: 201803HM001) in accordance with the Declaration of Helsinki. All participants signed a written consent before the experiment and declared to be free of injuries.

### Data collection

A cross-sectional study was designed to estimate the 1RM back squat based on GRF-derived variables during CMJ and SJ. The study comprised two parts: first, a 1RM back squat test; second, maximal vertical CMJ and SJ tests (randomized order) on a force plate. The maximal vertical jump tests were conducted within 2–7 days after the 1RM back squat test. Subsequently, the data were analyzed using stepwise linear regression models to develop estimation equations for the 1RM back squat based on CMJ and SJ performance. And the researcher was blinded to which participant the estimation was for.

Each participant initiated the study with a 1RM back squat test, using a barbell. This test followed a familiarization session and a standardized warm-up protocol that included both general warm-up and dynamic stretching routines. The test procedure adhered to the National Strength and Conditioning Association (NSCA) guidelines [46]: (1) commence with a light resistance, allowing participants to perform 5–10 repetitions easily, (2) rest for one minute, (3) gradually increase the weight by 10–20% and assess the participant’s 3–5RM, (4) rest for two minutes, (5) gradually increase the weight by 10–20% and assess the participant’s 2–3RM, (6) rest for 2–4 minutes, (7) gradually increase the weight by 10–20%, (8) instruct participants to aim for a 1RM, (9) if the attempt was successful, rest for 2–4 minutes and return to step 7; otherwise, rest for 2–4 minutes, reduce the weight by 5–10%, and repeat step 8, (10) continue adjusting the load until participants can successfully complete one repetition with proper technique within a maximum of five trials.

**Fig 1:**
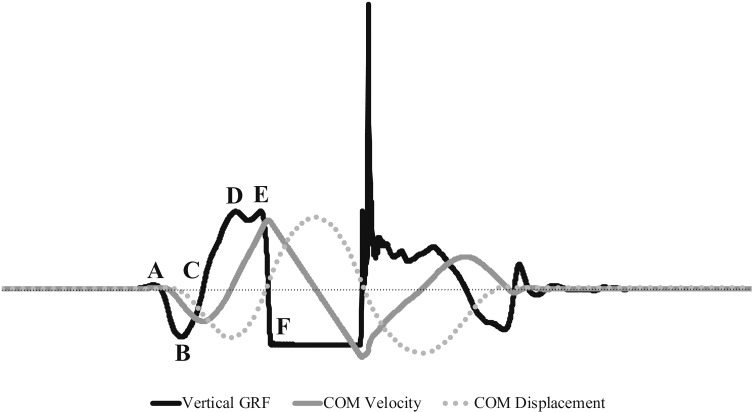
Vertical GRF force-time curve, COM vertical velocity, and COM vertical displacement of CMJ. Beginning to point A: weighting phase. Points A to C: unweighting phase. Points C to point D: braking phase. Points D to F: propulsion phase.

**Fig 2:**
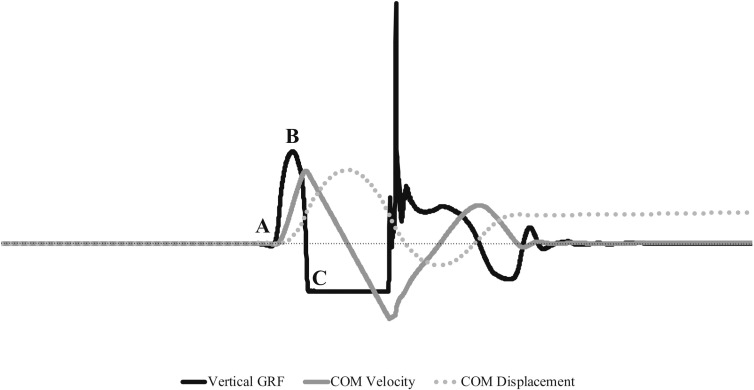
Vertical GRF force-time curve, COM vertical velocity, and COM vertical displacement of SJ. Beginning to Point A: weighting phase. Points A to C: propulsion phase.

During the maximal vertical jump test, all participants underwent a standardized warm-up procedure that included stretching, dynamic lower limb exercises, and jumps. Each participant completed three CMJ and three SJ in a random order, as outlined by Bender [18,19,47]. Participants were allowed to rest for three minutes between jumps. Before initiating each jump, participants were instructed to stand still for five seconds to prepare for the jump. During the jumps, participants were directed to keep their hands on their hips, jump as high as possible, and maintain a stationary stance for at least five seconds after landing, following previous guidelines [47]. GRF data were recorded and processed via a Kistler force plate model 9827 (Kistler Holding AG, Winterthur, Switzerland) (90x60 cm) at a sampling frequency of 1000 Hz (Figs 1 and 2) with BioWare software (Kistler Instruments Inc., Type 2812A, Version 5.4.3.0, Winterthur, Switzerland). The GRF-time profiles were analyzed to ensure compliance with the instructions. Trials were repeated if deviations from the instructions were observed. The highest CMJ and SJ values were selected for subsequent statistical analysis.

### Data processing

Figs 1 and 2 illustrate the phases and GRF variables and COM position of CMJ and SJ based on prior research [33,35,36,48–54]. These phases included:

1. Weighting phase: from beginning of the GRF data collection to the initial start of the vertical jump movement (CMJ and SJ: from the start of GRF data collection to point A)
2. Unweighting phase: from the initial start of the vertical jump movement through to the instant at which force returns to BW (CMJ: points A to C)
3. Braking phase: from the instant of peak negative COM velocity through to when COM velocity increases to zero (CMJ: points C to D)
4. Propulsion phase: starts when a positive COM velocity is achieved and continues through to the instant of take-off (CMJ: points D to F; SJ: points A to C)

Body weight was determined during the first 500 data points of vertical GRF during the weighting phase [35]. The start of the jump was defined as the time point 30 ms before the vertical GRF exceeded the threshold (the total system weight ±  5 SD). Take-off was identified as the moment when the vertical GRF decreased to <  20 N after the start of the propulsion phase [40]. The selected GRF variables included peak and mean power, relative net impulse, jump height, and peak kinetic energy.

The following definitions clarify the variables:

- Peak power: The maximum power from the unweighting to the propulsion phases in CMJ and during the propulsion phase in SJ
- Mean power: The average power from the unweighting to the propulsion phases in CMJ and during the propulsion phase in SJ
- Relative net impulse: The total impulse from the unweighting to the propulsion phases/ jumper’s body mass in CMJ and during the propulsion phase/ jumper’s body mass in SJ
- Jump height: Calculated using the equation: JH =  V_TO_^2^/ 2g (where V_TO_ =  COM vertical velocity at take-off and g is the acceleration due to gravity)
- Peak kinetic energy: Calculated as PKE =  1/2 ${\text{mv}}_{\text{Peak}}^{2}$ (where m =  body mass and V_Peak_ =  Peak COM vertical velocity: The maximum vertical velocity of the COM from the unweighting to the propulsion phases in CMJ and during the propulsion phase in SJ)

Previous studies showed that these variables respond to training interventions [17,55–58], which underlines the practical relevance of including those variables in the current investigation.

### Statistical analyses

After obtaining and processing GRF via BioWare software, data were merged and organized via Microsoft Excel for further analysis. Statistical analysis was conducted via SPSS software version 23 (IBM Corp., Armonk, NY, USA). The Shapiro-Wilk test was conducted to assess the normality of each variable. To determine the reliability of the vertical jumps, a test-retest reliability analysis was performed using a 2-way random (type, absolute agreement) intraclass correlation coefficient (ICC) calculated for variables recorded during the separate testing sessions. ICC values of (ICC <  0.5), (0.5 ≤  ICC <  0.75), (0.75 ≤  ICC <  0.9) and (ICC ≥  0.9) were interpreted as poor, moderate, good and excellent respectively, based on the lower bound of the 95% confidence interval [59]. The standard error of measurement (SEM) was also calculated as SEM =  SD ×  $\sqrt{1-ICC}$ [60]. The percentage of SEM (%) was calculated as SEM (%) =  $\left(SEM/Mean\right)\times 100$ [61]. The mean represents the average GRF variable values across three trials. Pearson product-moment correlations were calculated between the GRF variables and 1RM back squat derived from the vertical jumps including CMJ and SJ. Correlation coefficients were considered trivial (*r* <  0.1), small (0.1 ≤  *r* <  0.3), moderate (0.3 ≤  *r* <  0.5), high (0.5 ≤  *r* <  0.7), very high (0.7 ≤  *r* <  0.9), or extremely high (*r* ≥  0.9) [62]. GRF-derived variables were used to develop the estimation equation for 1RM back squat via stepwise linear regression analysis. Collinearity among predictors was assessed, and co-correlating GRF-derived variables have been removed from the regression analysis. Paired samples t-test was used to examine the differences between the estimated 1RM back squat (1RM_e_) and the measured 1RM back squat (1RM_m_). The effect size of the differences between the 1RM_e_ and the 1RM_m_ was calculated as Cohen’s *d*. The magnitude of Cohen’s *d* was interpreted as negligible (*d* <  0.2), small (0.2 ≤  *d* <  0.5), moderate (0.5 ≤  *d* <  0.8), or large (*d* ≥  0.8) [63,64]. Standard error of estimate was calculated as SEE =  $\sqrt{\sum {\left(1RMe-1RMm\right)}^{2}/\left(N-2\right)}$, where N represented the number of the participants. Bland-Altman plots depicted the agreement between 1RM_e_ and 1RM_m_ [45]. Effect sizes for correlation, regression, and t-tests were presented as *r,* adjusted *R*², and *d*, respectively. Statistical significance level was set at *p* < .05. Post-hoc Bonferroni correction was applied to correlation analysis to avoid the accumulation of family-wise error rates due to multiple correlation tests.

## Results

### Reliability of CMJ and SJ measurements

Table 1 displays ICC and *SEM* values calculated for the entire sample size to quantify the relationship between the GRF variables achieved during three CMJ and SJ trials.

**Table 1:** Reliability statistics for the GRF variables derived from CMJ and SJ.

GRF variables	CMJ		SJ	
	ICC (95% CI)	SEM	ICC (95% CI)	SEM
Peak power (W)	.97 (.93–.99)	219.64 (5%)	.99 (.97–1.00)	129.36 (3%)
Mean power (W)	.96 (.91–.99)	29.08 (7%)	.98 (.96–.99)	61.29 (5%)
Relative net impulse (N·s·kg^-1^)	.98 (.94–.99)	0.06 (2%)	.97 (.91–.99)	0.07 (3%)
Jump height (cm)	.97 (.93–.99)	1.77 (5%)	.97 (.93–.99)	1.63 (6%)
Peak kinetic energy (J)	.96 (.89–.99)	22.15 (7%)	.98 (.95–.99)	12.31 (5%)

ICC =  intraclass correlation coefficient; CI =  confidence interval

### Correlation between GRF-derived variables and measured 1RM back squat

The mean 1RM_m_ across all participants was 1.7 ±  0.4 times their body weight (BW). Table 2 shows the results of the correlation between GRF-derived variables and 1RM_m_ for all participants. Five out of 5 variables in CMJ and SJ showed significant and very high correlations with 1RM (*r* = .88–.95, *p* < .001 and *r* = .76–.90, *p* < .05, respectively).

**Table 2:** Correlation between GRF-derived variables and 1RM back squat in CMJ and SJ.

GRF variables	CMJ			SJ		
	Mean ± SD	r	p	Mean ± SD	r	p
Peak power (W)	4336.2 ± 1341.9	.93	**	4006.3 ± 1307.6	.90	**
Mean power (W)	433.9 ± 164.7	.88	**	1435 ± 485	.90	**
Relative net impulse (N·s·kg^-1^)	2.9 ± 0.4	.90	**	2.6 ± 0.4	.77	*
Jump height (cm)	39.9 ± 11.5	.89	**	30.6 ± 9.8	.76	*
Peak kinetic energy (J)	315.7 ± 113.1	.95	**	245 ± 92.9	.86	**

CMJ=countermovement jump; SJ=squat jump;

*p < .05

**p < .001

### 1RM back squat estimation equation using GRF-derived variables from CMJ

**Fig 3:**
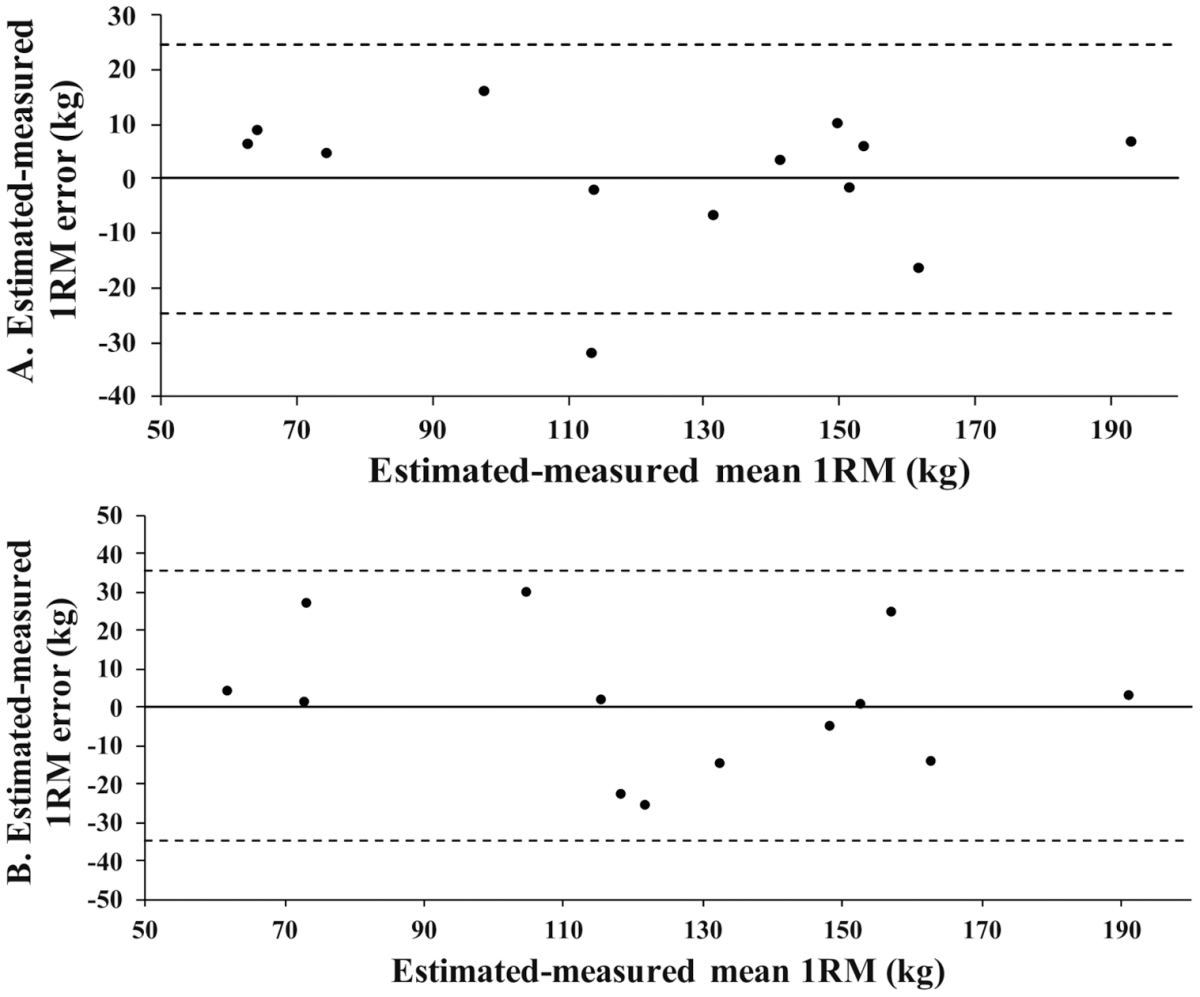
A. Bland-Altman plots between the 1RMe and 1RMm using GRF variables from CMJ. B. Bland-Altman plots between the 1RM_e_ and 1RM_m_ using GRF variables from SJ. Dashed line represents 95% confidence interval.

The following estimation equations were derived from GRF during CMJ: 1RM =  0.352 ×  peak kinetic energy +  12.775 (adjusted *R*^*2*^ = .90, *p* < .001, standard error of the estimate [*SEE*] =  13.24 kg, mean error =  7.4% of mean 1RM_m_). Paired samples *t-*tests on the validation sample showed no significant (*p = * 1.00, *d* <  0.01) difference between the 1RM_e_ (123.9 ±  39.8 kg) and 1RM_m_ (123.9 ±  41.8 kg) (Tables 3 and 4). The Bland-Altman plot showing 95% limits of agreement (LOA) between 1RM_e_ and 1RM_m_ was depicted in Fig 3A. The 95% LOA ranged from -24.87 to 24.82 kg between the 1RM_e_ and 1RM_m_ in CMJ. The post-hoc analysis revealed that the power of the estimation model derived from CMJ for 1RM was 1.

**Table 3:** Mean, standard deviations, and correlations of 1RM_m_, 1RM_e_ of CMJ, and 1RM_e_ of SJ.

Mean ± SD			Mean SE			*r*; *p*	
1RMm	1RMeC	1RMeS	1RMm	1RMeC	1RMeS	1RMm - 1RMeC	1RMm - 1RMeS
123.9 ± 41.8	123.9 ± 39.8	124.2 ± 37.8	11.6	11	10.5	.95; < .001	.90; < .001

1RM_m_ = 1RM actually measured; 1RM_e_^C^ = 1RM estimated from CMJ; 1RM_e_^S^ = 1RM estimated from SJ.

**Table 4:** Paired samples t tests of 1RM_m_, 1RM_e_ of CMJ, and 1RM_e_ of SJ.

	95% CI						
	Mean	SD	Mean SE	Lower	Upper	*p*	*d*
**1RM**_**m**_ **- 1RM**_**e**_^**C**^							
1RM = 0.352 peak kinetic energy + 12.775	.02	12.68	3.52	-7.64	7.68	1.00	<0.01
**1RM**_**m**_ **- 1RM**_**e**_^**S**^							
1RM = 0.078 mean power + 12.254	-.26	17.93	4.97	-11.1	10.57	.96	-0.02

1RM_m_ = 1RM actually measured; 1RM_e_^C^ = 1RM estimated from CMJ; 1RM_e_^S^ = 1RM estimated from SJ.

### 1RM back squat estimation equation using GRF-derived variables from SJ

The following estimation equations were derived from GRF during SJ: 1RM =  0.078 ×  mean power +  12.254 (adjusted *R*^*2*^ = .80, *p* < .001, *SEE* =  18.72 kg, mean error =  10.7% of mean 1RM_m_). Paired samples *t-*tests on the validation sample showed no significant (*p = *.96, *d* =  -0.02) difference between the 1RM_e_ (124.2 ±  37.8 kg) and 1RM_m_ (123.9 ±  41.8 kg) (Tables 3 and 4). The Bland-Altman plot, which includes the 95%, is depicted in Fig. 3B. LOA ranged from -34.9 to 35.4 kg between the 1RM_e_ and 1RM_m_ in SJ. The post-hoc analysis revealed that the power of the estimation model derived from SJ for 1RM was 1.

## Discussion

The objective of this study was to develop equations for estimating 1RM back squat based on GRF during CMJ and SJ. Key findings were: (1) Measurements of GRF variables during vertical jumps, including CMJ and SJ, are both reliable assessments; (2) the GRF variables derived from vertical jumps were significantly correlated with the 1RM back squat; (3) the estimation models of 1RM back squat based on GRF variables were developed across two jump types explaining 80%-90% of the total variance in the 1RM back squat, with an error range of 7.4–10.7% of the mean 1RM_m_.

All of the GRF variables in our study, derived from healthy adults with regular exercise habits during vertical jumps, showed good to excellent reliability (Table 1). This indicates that the measurement error is limited, being less than the individual variability. Our ICC results (i.e., 0.96–0.98 in CMJ and 0.97–0.99 in SJ) were within previously reported ranges of ICC values (i.e., 0.65–0.99) in vertical jump kinetics and back squat performance [14,15,65–67]. In summary, the investigation of the GRF variables during vertical jump tests provides a reliable assessment tool for sports scientists, sports medicine professionals, coaches, and athletes.

Another current finding was the strong correlation between GRF-related variables during vertical jumps and the 1RM back squat, suggesting vertical jump performance as an indicator of maximal back squat strength. Specifically, the study highlighted peak kinetic energy, peak power, and relative net impulse in the CMJ, as well as peak power and mean power in the SJ. The previously documented relevance of peak movement velocity [68] and body mass [69] for 1RM back squat performance may explain the currently observed, strongest correlation between 1RM back squat and peak kinetic energy derived from body mass multiplied by the square of peak velocity. Supported by the previous reports on the role of relative net impulse in determining jump performance [39], impulse contributed to the current predictions. Notably, limited research explored the relationship between the 1RM back squat and GRF variables during CMJ and SJ. This study contributed to filling this gap and demonstrated that vertical jump kinetics can effectively reflect an individual’s maximal strength capabilities.

The estimation models for 1RM back squat based on GRF variables were developed using data from CMJ and SJ. The CMJ-based model demonstrated a higher *R*^*2*^ and smaller error range of the mean 1RM_m_ compared to the SJ-based model. These findings suggested that CMJ may be a more reliable predictor of 1RM back squat performance. The higher *R²* value implied that the CMJ-based model explained more of the variance in 1RM back squat performance, while the smaller error range indicated greater precision and consistency. One potential explanation for the superior performance of the CMJ-based model could be the involvement of the SSC in CMJ, which was less pronounced in SJ. The SSC is a natural muscle function that enhances force production and efficiency [18,70], potentially leading to better predictive validity for dynamic movements like the back squat. The elastic energy stored during the eccentric phase of the CMJ and released during the concentric phase is similar to that utilized in the back squat and might contribute to the higher predictive accuracy observed.

No studies have established estimation equations for lower body maximum strength through vertical jumping. However, several previous studies have developed estimation equations for lower body average and maximum power in vertical jumping [28,29,42,43]. These studies selected body mass and jump height to develop the estimation equations and the *R*^*2*^ values reported range from 0.74 to 0.93. Our results revealed adjusted *R*^2^ values of 0.90 and 0.80 for the estimation equations of 1RM back squat based on CMJ and SJ, respectively. This suggested that GRF variables from vertical jumps can be highly effective in estimating 1RM back squat strength. Furthermore, the observed error range in predicting 1RM back squat (13.2 to 18.7 kg) in our study aligned with the error ranges reported for estimating 1RM bench press (2 to 19 kg) using GRF data during a ballistic push-off [45,71]. This comparison indicates that the estimation equations we developed for back squat strength have comparable predictive accuracy to those used for bench press strength. In addition, the measurement error in other 1RM back squat estimations via individual load-velocity profiles during the back squat ranged from below 5 kg to 17.2 kg [72,73]. Therefore, the load-velocity methods may exhibit lower measurement errors than those reported in this study. However, these studies estimated 1RM based on load-velocity profiles from participants squatting at least 1.5 BW as their 1RM, which was 50% higher than in the current study. Therefore, it was unclear if estimation approaches were applicable across samples with different strength levels. In summary, these findings underscored the validity of using vertical jump metrics for estimating lower body strength and suggested that the errors in our predictions fall within a range similar to those observed in established methods. This alignment supports the reliability of our estimation equations and highlights their potential utility in accurately assessing lower body strength through vertical jump performance.

The current findings were limited to physically active participants who integrated the loaded back squats in their frequent training and were capable of squatting an extra weight of 1 BW or more. To generalize the estimation model, future research may recruit individuals with a larger variability regarding strength and skill levels. Second, although the sample size was sufficient to detect the desired effect sizes in the current study and aligned with recommendations from the literature [44,74], smaller effects may have been undetected. Studies that aim for assessing a larger range of effects in more diverse samples are recommended to recruite a larger sample. In the current study, as expected, effect sizes were so large that the acual observed power of 1−β =  1 was achieved via post-hoc analysis, suggesting a sufficient sample size for the current effects. Third, the small age range (22– 25 years) and the underrepresentation of females (23%) in the current sample. A previous study showed no significant impact of age and sex on the estimation of peak muscle power from the CMJ [28]. In addition, the current study did not attempt to investigate the effect of age and sex or to derive age- and sex-specific prediction models. Therefore, the limitation in sample characteristics was not considered consequential for practical implication. Another limitation was that the *SEE* rates reported in this study were based on a single assessment without cross-validation after a period of prescribed training. However, the accuracy of the estimation model still needs to be further improved in the future. Considering the current error rates, the proposed model may be more suitable for providing a general fitness assessment of lower limb strength than for prescribing exact training loads.

## Conclusion

This study presents evidence that GRF-derived variables obtained during CMJ and SJ may provide alternative methods for assessing the 1RM back squat. CMJ has been demonstrated to be a more accurate predictor of 1RM back squat compared to the SJ. This study developed a general equation using peak kinetic energy from CMJ data applicable to healthy young adults (*r* = .95, adjusted *R*^*2*^ = .90, mean error =  7.4% of the mean 1RM_m_). The proposed equations could be implemented to enhance their potential impact and relevance for practical monitoring in strength training. Incorporating lower body maximum strength assessment through GRF variables from vertical jumps has the potential to improve general fitness programs and serve as a valuable component of periodized strength and conditioning protocols for both general populations and athletes. Furthermore, the developed estimation models for 1RM back squat based on GRF-derived variables from vertical jumps could be utilized to create a force assessment algorithm for sports technology companies.

## Supporting information

S1 Table Raw data from all participants. (XLSX)
